# Osteolytic bone lesions as an initial presenting manifestation of adult acute lymphoblastic leukemia: a mini review

**DOI:** 10.1097/MS9.0000000000001065

**Published:** 2023-07-20

**Authors:** Abdulrahman F. Al-Mashdali, Hussam N. Al-Dubai, Mohamed A. Yassin

**Affiliations:** a Department of Oncology, Hematology and BMT Section, National Center for Cancer Care and Research, Hamad Medical Corporation; b Department of Internal Medicine, Hamad Medical Corporation, Doha, Qatar

**Keywords:** acute lymphoblastic leukemia, adult, ALL, complications, osteolytic bone lesions

## Abstract

Hematological malignancies can lead to bone lesions, and the most common example is the osteolytic lesions found in multiple myeloma. Cases of osteolytic lesions have been rarely reported in acute lymphoblastic leukemia (ALL), non-Hodgkin lymphoma, Waldenström macroglobulinemia, chronic lymphocytic leukemia, acute myeloid leukemia, and myeloproliferative neoplasms. This review sheds light on the association between ALL and osteolytic bone lesions. To our knowledge, we found 15 cases of patients with ALL who developed osteolytic lesions. Most patients were males with a median age of 29 years. B-cell ALL was the most common type of ALL associated with osteolytic lesions. All patients presented with bone pain, and hypercalcemia was found in 80% of the reported cases. Osteolytic lesions were detected by plain radiography (X-ray) in approximately half of the patients; computed tomography, MRI, or PET scans confirmed the osteolytic lesions in the remaining patients. The axial skeleton was mainly affected. Based on our review, there was no association between osteolytic bone lesions and the Philadelphia chromosome. There are no case of spinal cord compression in adults ALL patients attributed to osteolytic lesions of the vertebra. The majority of patients received chemotherapy, and the outcomes among these patients were variable. Almost all of them achieved complete remission. However, two patients developed a disease relapse. Given that our review is solely based on case reports, we could not conclude if the presence of osteolytic bone lesions is a prognostic factor for adverse outcomes or indicates an ‘aggressive’ form of ALL.

## Introduction

Highlights

- Osteolytic lesions are an infrequent finding in patients with acute lymphoblastic leukemia (ALL).
- Most patients with ALL who develop osteolytic lesions are male.
- Bone pain is the main presenting symptom in these patients, and the majority of them have hypercalcemia also.
- The presence of osteolytic lesions could not be considered to indicate a poor prognosis in patients with ALL.

Acute lymphoblastic leukemia (ALL) is a malignancy of B or T lymphoblasts characterized by uncontrolled proliferation of abnormal, immature lymphocytes and their progenitors, which ultimately leads to the replacement of bone marrow elements and infiltration of extramedullary sites^1^. ALL represents around 12% of all leukemia cases. ALL occurs slightly more frequently in males than females and more frequently in White people than Black^2^. Less than 20% of ALL cases occur in adults. B-cell ALL (B-ALL) accounts for more than 75% of cases, and T-cell ALL comprises the remaining cases^3^. The clinical presentation of ALL is nonspecific, including fatigue (due to anemia), bleeding tendency or easy bruising (due to thrombocytopenia), and infections (due to neutropenia). Involvement of extramedullary organs, including lymphadenopathy, splenomegaly, or hepatomegaly, occurs in around 50% of cases^4^. In addition, some patients with ALL can present solely with musculoskeletal pain. The bone pain in ALL patients might be attributed to the excess number of leukemic cells in the medullary canal of the bone, causing a mass-like effect with periosteal stretching^5^.

Hematological malignancies can lead to bone lesions, most commonly the osteolytic lesions found in multiple myeloma (MM). Cases of osteolytic lesions have been rarely reported in ALL, non-Hodgkin lymphoma, Waldenström macroglobulinemia, chronic lymphocytic leukemia, acute myeloid leukemia, and myeloproliferative neoplasms^6^. Rarely, osteolytic bone lesions in adult ALL patients can cause bone pain or even lead to pathological fracture^5^. In a study of musculoskeletal manifestations in pediatric acute leukemia, ~13% of the cases had osteolytic bone lesions, most of which were diagnosed with ALL^7^. However, the prevalence of osteolytic lesions in adult ALL is unknown, and only a handful of cases are found in the literature. This review aims to shed light on the association between adult ALL and osteolytic lesions.

## Methods

A literature search was performed for English language articles using PubMed, Scopus, and Google Scholar, for any date up to August 2022. The following keywords were used ‘ALL’, ‘acute lymphoblastic leukemia’, ‘osteolytic bone lesions’, and ‘osteolysis’. Search results were initially reviewed by title and abstract, and articles were selected for more in-depth analysis if deemed relevant. Pertinent articles were examined in-depth for this report.

## Results

Tables 1 and 2 summarizes the clinical information of the patients involved in our review. We found 15 cases of patients with ALL who presented with osteolytic lesions. They were reported in 14 articles between April 1998 and February 2022^5,8–20^. Most patients were males (12 out of 15). The median age was 29 years (ranging from 22 to 78 years), with most patients being young (between 20 and 30 years old).

**Table 1:** Summary of the reported cases

Case number	Author (year)	Age/Sex/Ethnicity	Initial presentation	Blood count	Ca	Hypercalcemia management	BM	Dx	Karyotype	Philadelphia	Imaging
1	Antunovic *et al*. (1998)^8^	24/M N/A	Bone pain, weakness, headache, vomiting	WBC 24 Hb 6 Plt 71 Blast 22%	High	Hydration, diuretics, and pamidronate	85% blasts	T-ALL	N	Negative	XR: multiple lytic lesions in the skull, pelvis, and long bones
2	Fukasawa *et al*. (2001)^9^	53/F N/A	Nausea, drowsiness, bone pain	WBC N Hb 7.7 Plt N No blast	High	None	94% blasts	B-ALL	N	Negative	XR: multiple osteolytic lesions in the skull and ribs
3	Chung *et al*. (2011)^10^	35/M Korean	Jaw pain, fatigue, fever	WBC 10 Hb N/A Plt 261 Blast N/A	High	Hemodialysis, calcitonin, and risedronate	80% blasts	B-ALL	46,XY,der(1)add(1)(q25)del(1)(p31),der(7)t(1;7)(p13;q22)[15]/46,XY[8]	Negative	XR: osteolytic lesion on the left mandibular body and ascending ramus area
4	Verma *et al*. (2014)^11^	27/F N/A	Back pain, bilateral lower limbs weakness, numbness	WBC 2 Hb 8 Plt 50 Blast 12%	N	None	92% blasts	Pre B-ALL	N/A	N/A	XR: multiple osteolytic lesions in the skull
5	Kaiafa *et al*. (2015)^12^	24/M N/A	Lumbar pain	WBC 8.8 Hb 17.4 Plt 130 No blast	High	Hydration, furosemide, corticoids, and bisphosphonates	9% blasts	Pre B-ALL	46,XY,dup(1)(q21q32),del(8)(p22) [12]/46,XY[8]	Negative	CT: multiple diffused well-defined osteolytic lesions in all lumbar vertebrae, sacrum, ilium, and both femora
6	Virijević *et al*. (2016)^13^	28/M N/A	Exhaustion, weight loss, fever, bone pain	WBC 3 Hb 10.7 Plt 24 No blast	High	Furosemide and corticosteroids	70% blasts	B-ALL	N	Negative	XR: extensive osteolytic lesions involving the skull, pelvis, ribs, vertebra, and long bones
7	Naaraayan *et al*. (2016)^14^	29/M N/A	Nausea, vomiting, fatigue, right back and hip pain	WBC 6.1 Hb 11.9 Plt 128 Blast N/A	High	Hydration	90–100% blasts	B-ALL	N/A	N/A	CT: innumerable, punched-out, osteolytic lesions in the axial skeleton and the extremities. Pathologic fractures of multiple ribs
8	Granacher *et al*. (2017)^15^	34/M N/A	Back pain	WBC N Hb N Plt 79 No blasts	High	Corticosteroids and zoledronic acid	>50% blasts	Pre B-ALL	t(9,22)(q34,q11,2)	Positive	XR: multiple osteolytic bone lesions of the ribs and lumbar spine
9	Mahmood *et al*. (2017)^16^	22/M N/A	Limbs pain, proximal muscle weakness, fever, increased volume and frequency of urine	WBC 8.6 Hb 6.2 Plt 223 No blast	High	Hydration, furosemide, and ibandronate	31% blasts	Pre B-ALL	N/A	N/A	XR: multiple osteolytic lesions in the iliac bones, skull, and the axial skeleton
10	Angsubhakorn *et al*. (2018)^5^	37/M N/A	Back pain, difficulty walking	WBC 6.1 Hb 7.5 Plt 291 Blast N/A	N	None	80% blasts	B-ALL	N	Negative	MRI: multiple osteolytic lesions involving the thoracic and lumbar spine
11	El-Ashwah *et al*. (2018)^17^	28/M N/A	Generalized bone pains, fever, weight loss	WBC 9.2 Hb 9.2 Plt 194 No blast	High	Hydration, furosemide, and dexamethasone	95% blasts	B-ALL	N	Negative	CT: multiple osteolytic bone lesions affecting the dorsal, lumbar spine, symphysis pubis, iliac, pubic bones, and sternum
12	El-Ashwah *et al*. (2018)^17^	27/F N/A	Vomiting, frequent urination, generalized bone ache	WBC 7.3 Hb 8.9 Plt 140 No blast	High	Hydration, furosemide, and dexamethasone	95% blasts	B-ALL	N/A	N/A	CT: multiple osteolytic lesions affecting both iliac bones, sacrum, vertebra, sternum, and skull
13	Chhakchhuak *et al*. (2020)^18^	32/M N/A	Weakness, back pain, fever, nocturia	WBC N/A Hb 10 Plt N/A Blast 29%	High	Hydration, zoledronate, and calcitonin	79% blasts	B-ALL	N/A	N/A	XR: multiple osteolytic lesions in the skull
14	Kim *et al*. (2021)^19^	78/M N/A	Back pain	WBC 6.06 Hb 10.2 Plt 132 No blast	N	None	70.4% blasts	B-ALL	N	Negative	CT: multiple osteolytic lesions in the skull, vertebrae, rib, and both pelvic bones
15	Habib *et al*. (2022)^20^	29/M N/A	Back pain, limping	WBC 7.5 Hb 10 Plt 157 No blast	High	Steroids	36% blasts	Pre B-ALL	N/A	N/A	CT: diffuse lytic skeletal infiltration involving sternum, ribs, vertebral column, and pelvic bones

BM, bone marrow; Ca, calcium; CT, computed tomography; Dx, diagnosis; F, female; Hb, hemoglobin; N, normal; N/A, not available; M, male; MRI, magnetic resonance imaging; Plt, platelets; Pre, precursor; WBC, white blood cell; XR, X-ray.

**Table 2:** Summary of the reported cases (continued)

Patient number	Management	Outcome
1	Induction: LALA 94 protocol Consolidation: mitoxantrone and cytosine arabinoside, and later with a high dose of methotrexate and folinic acid rescue	Ten months after chemotherapy, he was still in remission, his calcium was normal, and his bone pain resolved. Osteolytic bone lesions remained unchanged
2	Induction: modified JALSG-ALL 90 protocol	Remission after 2 weeks of treatment. 26 months after the chemotherapy, the patient was still in complete remission, and the Ca level was within normal range. Osteolytic lesions in the skull and ribs were still present
3	None	Died of acute pneumonia
4	Induction: GMALL protocol with triple intrathecal therapy	LL (lower limb) weakness improved over 2 weeks. Remission after induction
5	Initial: Hyper-CVAD (8 cycles) with intrathecal infusions of alternately methotrexate and cytarabine Rescue: FLAG-IDA	Relapsed 23 months after the initial remission. Relapsed again 1 month after rescue therapy and died due to severe sepsis
6	Induction: Hyper-CVAD Salvage: FLAG-IDA Allogeneic stem cell transplantation	Remission after stem cell transplant. Relapsed 6 months after remission and died
7	N/A	Succumbed to progressive leukemia a year later
8	Induction: prednisolone, daunorubicin, vincristine, and PEG-l-asparaginase with prophylactic intrathecal methotrexate and dexamethasone	Achieved complete molecular remission after the first induction
9	N/A	N/A
10	Induction: Hyper-CVAD (4 cycles)	Achieved complete response. Planned for allogeneic stem cell transplantation
11	MRC-UK protocol Salvage: HAM protocol	Complete hematological response after phase I. Relapse after 1.5 years, for which he received salvage, with complete remission. After 1 year, he developed pancytopenia, pneumonia, and septic shock. The outcome was death
12	Induction: Hyper-CVAD chemotherapy protocol with triple intrathecal twice weekly and cranial irradiation	Developed septic shock during induction and died
13	N/A	N/A
14	Radiation	Received radiation therapy because of his poor general condition
15	BFM protocol	N/A

N/A, not available.

B-ALL was the most common ALL associated with osteolytic lesions (14 cases), and T-ALL was diagnosed in only one case. The Philadelphia chromosome was detected in only one patient (patient number 8), and he received TKI (tyrosine kinase inhibitor) (imatinib) before starting induction chemotherapy. In 80% of patients, hypercalcemia was managed mainly with hydration, furosemide, and corticosteroids. Extramedullary involvement was not reported in most cases, except for two patients (patients 4 and 12), in whom evidence of central nervous system (CNS) involvement was reported.

All patients presented with bone pain. Other common symptoms at the presentation were fatigue, weight loss, and fever. X-ray detected osteolytic lesions in approximately half of the patients. The osteolytic lesions were detected in the remaining patients by computed tomography (CT), MRI, or PET scans. In almost all patients, bone lesions were present at the time of diagnosis with ALL, except for one patient (patient 5) in whom bone lesions developed when he had a disease relapse. The most affected bones were the vertebrae, the skull, and the pelvis. Ribs, sternum, and long bones were less frequently involved. Interestingly, one patient (patient 3) only had lytic lesions involving his jaw. Majority of patients did not receive specific treatment for the osteolytic lesions. Only one patient (patient 14) received local radiotherapy to the thoracic and lumbar spines.

Most patients received chemotherapy for the treatment of ALL. Different chemotherapy regimens were given, as shown in Table 2. The outcomes among these patients were variable. Almost all of them achieved complete remission. However, two patients developed a disease relapse. Despite the improvement of bone pain with chemotherapy, the persistence of osteolytic lesions was confirmed in some patients. Measurable residual disease (MRD) negativity was not reported in all cases. Only one patient (patient 6) underwent allogeneic stem cell transplantation, which reduced the osteolytic lesions. Nevertheless, he relapsed after 6 months and died. Two patients were not treated with chemotherapy. One of them (patient 3) died from severe pneumonia before starting chemotherapy, and the other one (patient 14) opted for conservative treatment because of his poor general condition, for which he received radiation therapy only.

## Discussion

ALL is a malignant proliferation of lymphoid progenitor cells in the bone marrow and extramedullary sites. The incidence of ALL is bimodal, with the first peak occurring in childhood and the second peak of incidence occurring around the age of 60. Though 80% of ALL occurs in the pediatric population, the prognosis in adults is much worse^2^. Diagnosis of ALL is made by the presence of 20% or more lymphoblasts in the bone marrow or peripheral blood. Evaluation for morphology, flow cytometry, immunophenotyping, and cytogenetic testing is important for confirming the diagnosis and risk stratification. Given that 5–8% of ALL patients will have evidence of CNS involvement on initial presentation, lumbar puncture with CSF (cerebrospinal fluid) analysis should be done in all patients at the time of diagnosis, even in the absence of neurological abnormality^3^.

Bone is a common site of metastases in cancer patients. Prostate and breast cancers are responsible for more than two-thirds of bone metastasis. The clinical manifestations of bone metastasis include severe pain, impaired mobility, pathological fractures, spinal cord compression, and hypercalcemia^21^. Bone metastases are categorized as osteolytic, osteoblastic, or mixed based on the primary interference mechanism with bone remodeling. The destruction of normal bone characterizes osteolytic bone metastasis, occurring in MM, renal cell carcinoma, melanoma, non-small cell lung cancer, non-Hodgkin lymphoma, thyroid cancer, or Langerhans-cell histiocytosis^22^. Different imaging modalities can be used for the evaluation of osteolytic bone lesions, including plain radiography with a sensitivity of 44–50%, CT scans with a sensitivity of 71–100%, MRI with a sensitivity of 82–100%, and PET with a sensitivity of more than 90%^22^.

Osteolytic metastasis from ALL in adults is a rare condition. The exact pathophysiology of osteolysis in ALL patients is unknown. However, serum from ALL patients with osteolysis can induce in-vitro proliferation of osteoclasts, indicating the mechanism of lymphoblasts induces osteoclasts activation in the body. PTHrP (parathyroid hormone-related protein) secretion by lymphoblasts is the primary cause of osteolysis in ALL patients^23^; on the other hand, inflammatory cytokines excreted by lymphoblasts might be the contributing factors in a patient with normal serum PTHrP level^9^. The most important differential diagnosis for ALL with osteolytic bone lesions is MM, which should be ruled out in such patients. In a retrospective study of skeletal manifestations of pediatric ALL, the authors concluded that patients with osteolytic bone lesions might have a better survival rate attributed to better steroid responsiveness^7^. However, this correlation in adult patients with ALL is still not studied.

Our review revealed that no association between osteolytic bone lesions and the Philadelphia chromosome was detected in only one patient. Osteolytic lesions in adult ALL patients mainly present with bone pain. As the pathological fracture of the vertebral body leading to spinal cord compression is a complication of osteolytic lesions in patients with MM, there is no case of spinal cord compression in ALL patients attributed to osteolytic lesions of the vertebra. This indicates that the prognosis of osteolytic lesions in adult ALL might be better than MM.

In our center, there was a report of a 29‐year‐old male who presented with chronic back pain and progressive limping. He was diagnosed with B-ALL complicated by vertebral osteolytic lesions-ray spine was sufficient to pick the osteolytic lesions, and the MRI spine showed reduced height of multiple vertebral bodies with no spinal cord compression. Our patient's condition improved dramatically with steroid and chemotherapy and no radiotherapy was required. Similarly, most of the reported cases responded to chemotherapy and steroid therapy.

Given that our review is entirely based on a limited number of case reports, we could not conclude if the presence of osteolytic bone lesions is a prognostic factor for adverse outcomes or indicates an ‘aggressive’ form of leukemia in adult patients with ALL. We believe that further research is needed for a better understanding of the optimal management and prognosis of osteolytic lesions in adult ALL.

## Conclusion

Osteolytic lesions in adult patients with ALL are an infrequent finding and mainly occur in young adults. Plain radiography is often sufficient in diagnosing osteolytic lesions; however, other imaging modalities are occasionally required. Steroids and chemotherapy are sufficient for the symptomatic treatment of osteolytic lesions in most cases. Based on our review, the osteolytic lesions might not indicate a poor prognosis in adult patients with ALL.

## Ethical approval

Not applicable.

## Consent

Not applicable.

## Sources of funding

Open access funding was provided by Qatar National Library. It had no role in the collection, analysis and interpretation of data; in the writing of the manuscript; and in the decision to submit the manuscript for publication.

## Author contribution

A.F.A.-M. and M.A.Y.: conceptualization; A.F.A.-M. and H.N.A.-D.: data curation and literature review; A.F.A.-M. and H.N.A.-D.: writing – original draft; A.F.A.-M. and M.A.Y.: writing – review and editing.

## Conflicts of interest disclosure

The authors have no conflicts of interest to declare.

## Provenance and peer review

Not commissioned, externally peer-reviewed.

## Guarantor

Abdulrahman F. Al-Mashdali.
